# Behavioral Adjustments by a Small Neotropical Primate (*Callithrix jacchus*) in a Semiarid Caatinga Environment

**DOI:** 10.1155/2014/326524

**Published:** 2014-11-06

**Authors:** María Fernanda Castellón De la Fuente, Antonio Souto, Marilian Boachá Sampaio, Nicola Schiel

**Affiliations:** ^1^Departamento de Biologia, Universidade Federal Rural de Pernambuco, Rua Dom Manoel de Medeiros, s/n, Dois Irmãos, 52171-900 Recife, PE, Brazil; ^2^Departamento de Zoologia, Universidade Federal de Pernambuco, Avenida Prof. Moraes Rego, No. 1235, Cidade Universitária, 50670-901 Recife, PE, Brazil

## Abstract

We provide the first information on the behavior of a small primate (*Callithrix jacchus*) inhabiting a semiarid Caatinga environment in northeastern Brazil. We observed behavioral variations in response to temperature fluctuation throughout the day. Due to the high temperatures, low precipitation, and resource scarcity in the Caatinga, as well as the lack of physiological adaptations (e.g., a highly concentrated urine and a carotid rete to cool down the brain) of these primates, we expected that the common marmosets would exhibit behavioral adjustments, such as a prolonged resting period or the use of a large home range. During the six-month period, we collected 246 hours of behavioral data of two groups (10 individuals) of *Callithrix jacchus*. Most of the observed behavioral patterns were influenced by temperature fluctuation. Animals rested longer and reduced other activities, such as foraging, when temperatures were higher. Both study groups exploited home ranges of 2.21–3.26 ha, which is within the range described for common marmosets inhabiting the Atlantic Forest. Our findings confirm that common marmosets inhabiting the Caatinga adjust their behavioral patterns to cope with the high temperatures that characterize this environment and highlight their ability to survive across a wide range of different environmental conditions.

## 1. Introduction

Common marmosets (*Callithrix jacchus*) are distributed across northeastern Brazil and exploit a range of forest types including the humid Atlantic Forest and the semiarid Caatinga scrublands [1]. The Atlantic Forest is a moist tropical forest receiving more than 2000 mm of rain a year and contains high biological diversity [2]. Mean temperatures range between 14 and 21°C, reaching a maximum of 35°C [3]. The Caatinga, in contrast, is a mosaic of scrubs and patches of seasonally dry forest with temperatures reaching up to 40°C [4]; it receives approximately 500 mm of rain per year [4, 5] and has a more limited biodiversity, particularly in terms of the mammalian fauna [6].

Virtually all ecological and behavioral studies of wild groups of* Callithrix jacchus *have been conducted in the Atlantic Forest (e.g., [7–16]). By contrast so far only three studies were done with* C. jacchus *living in the Caatinga [17–19]. Moura [17] reported that, in the Caatinga,* C. jacchus* occurs in lower densities and smaller group sizes (average of 2.9 ± SD 1.67 individuals/group) than in areas of the Atlantic Forest (8.7 individuals/group). de Freitas et al. [18] refer to* C. jacchus* in the Caatinga as relatively abundant (169.7 and 116.7 individuals/km^2^). Amora et al. [19] reported on the consumption of new food items (e.g., parts of cactus species (flowers and fruits), nectar of a terrestrial bromeliad (*Encholirium spectabile*), leaves from seven different tree species, and the use of Capparaceae, Celastraceae, and Vitaceae).

Although the recent findings on common marmosets inhabiting the Caatinga are of value, broader, systematic investigations on their behavior in this environment are lacking. This is particularly important because studies suggest that mammals of the Caatinga lack pronounced physiological adaptations [20], a factor that makes behavioral adjustments and the presence of mesic refugia essential ([20]; e.g., [21]). It is worth noting that no primate (with the exception of the lorisids [22]) possesses the carotid rete to cool down the brain ([23]; e.g., [24, 25]).

The way behavior is affected by high temperatures in wild primates has been shown in different environments in baboons [24, 27, 28], chimpanzees [29, 30], howler monkeys [31], white-faced capuchins [32], spider monkeys [33, 34], brown lemurs [35], and vervet monkeys [36]. Previous results in semiarid environments are of particular interest for our study because of the usually higher temperatures that characterize such environments. In this regard, baboons inhabiting the savanna vegetation increased their resting period at high temperatures during the day [27, 28] and also displayed sand bathing behavior [24]. Similar to baboons, vervet monkeys inhabiting semiarid riparian woodland spent longer periods resting to cope with higher temperatures, even if this choice led to a reduced time of feeding [36].

So far all studies involving the effect of temperature in primate behavior were conducted with medium- to large-bodied primates. Having a larger body size means being better suited to cope with higher temperatures because of their relatively smaller body surface [37]. Thus, given the conditions and challenges of the Caatinga, that is, high temperatures and less available resources [4, 38], and due to the lack of physiological adaptations to live in hot environments and their small size, we expect that common marmosets will exhibit clear behavioral adjustments. More specifically, we expect to find (i) a prolonged resting period and (ii) the exploitation of a large home range to cope with the harsh conditions in the Caatinga.

Thus, the main goal of this research was to systematically observe how the behavior of common marmosets in the Caatinga environment varies in response to temperature fluctuation throughout the day. In addition, we also gathered more general information on their ecology and behavior (i.e., sleeping sites, sleeping cycle, and intergroup encounters), as we know very little about the way these small primates live in semiarid conditions. Finally, we compared, whenever possible, our findings with those reported in the literature for the Atlantic Forest common marmoset. This study will likely shed more light on the ecological success of common marmosets in different environments.

## 2. Materials and Methods

### 2.1. Study Site

**Figure 1:**
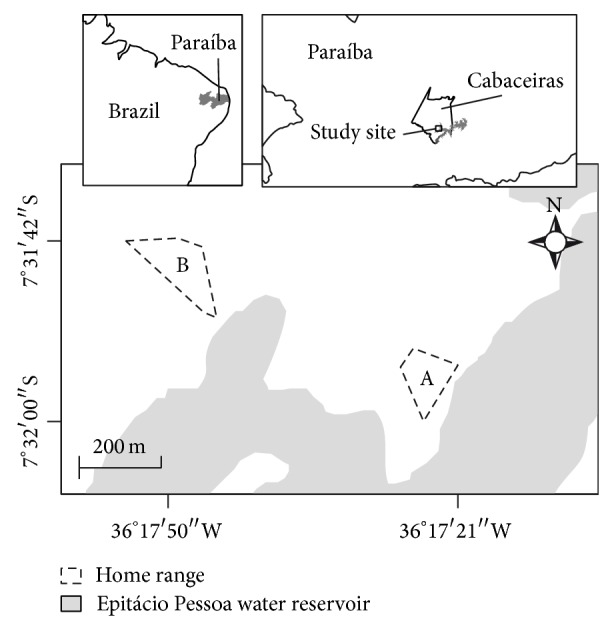
Study site at the Fazenda Marimbondo and home ranges of the wild* Callithrix jacchus* study groups (A and B) in the semiarid environment of Caatinga.

The study was conducted at the Fazenda Marimbondo, near the Municipality of Cabaceiras (384 m of altitude) in the state of Paraíba, northeastern Brazil (7°31′42′S and 36°17′50′W) (Figure 1). It covers an area of 400 ha in the microregion of eastern Cariri, which has a total area of 424.213 ha.

According to the Köppen climate classification, the study area is considered BSh type (hot semiarid) [39]. The mean maximum temperature during the study period was 34.1°C (highest: 36°C), mean minimum temperature 22.4°C (lowest: 20.4°C), mean lowest humidity 32.4%, and mean highest humidity 86.6% [40]. The temperatures were obtained from the INMET (National Institute for Meteorology; Cabaceiras station, ~5 km away from the study site) and refer to the time interval of 5 am to 5 pm, which corresponded to the time observational sessions were conducted (e.g., [32]). The mean annual rainfall over 86 years (1926 to 2011) was 336.6 mm [41], making the area one of the driest areas of Brazil. During the study period, precipitation levels were very low, averaging 10.7 mm/month [40]. The vegetation type is characterized by arboreal shrubs, typical of this semiarid region, and is dominated by a small number of scattered tree species (e.g., [4]). To measure the average vegetation density, we used the nearest neighbor method (for more details see Beasom and Haucke [42]; e.g., [13]). The vegetation is predominantly low (mean canopy height 3.55 ± SD 0.54 m) with a low tree/shrub density (4.460 individuals/ha; mean DBH (diameter at breast height) = 10.75 ± SD 2.97 cm); the mean distance between trees (105 ± SD 23.86 cm) is a result of limited rainfall and generally shallow and rocky soils with a low water retention capacity [43].

### 2.2. Subjects

For the present study, we observed two wild groups of* Callithrix jacchus *with a total of 16 individuals at the beginning of the study (Table 1). The composition of both groups changed over the course of the study: in group A, the breeding female and another adult female disappeared (January 2013), and a new female entered the group and became the primary breeder. In group B, four out of eight individuals disappeared (December 2012) overnight. In both groups, the animals were individually identified by using natural marks, sex, age, and social status within the group (e.g., [12, 16, 44]).

### 2.3. Procedure

After three months of habituation, systematic observations were performed by María Fernanda Castellón De la Fuente from November 2012 to April 2013, 10 days per month, for a total of 246 h of direct observation (146 h for group A and 100 h for group B). Quantitative behavioral data were collected using focal animal sampling methods [45, 46]. Each session consisted of a 10 min period of continuous observation and record of the behavioral patterns outlined in Table 2. During the 10 min session, when an animal was out of sight (“timeout” [47]) for more than 60 s, the session was stopped and discarded. The observed behavioral data were recorded using a voice recorder (Sony ICD-PX312; Sony Corporation, Tokyo).

Sessions were conducted between 5 am and 5 pm. The day was divided into 12 time intervals each corresponding to 1 h (e.g., 5 am-6 am; 6 am-7 am, etc.). We chose this method to be able to subsequently verify a possible correlation between the behavioral frequency and the temperature fluctuations among time intervals. Focal subjects were chosen randomly [47]. For each individual, two to four sessions per day were recorded. Individuals were observed for equal amounts of time (150 sessions per individual) except for the adult female that entered group A in January (126 sessions), for a total of 1476 sessions. We attempted to distribute the behavioral observations of each individual equally over all time intervals.

The home ranges were estimated by using the minimum convex polygon method [48]. We used a GPS (eTrex20; Garmin International Inc., Kansas) to mark every new location where individuals of each group were observed. Sleeping site locations were determined and tagged with the GPS when at the end of the day all animals of the group were gathered on a certain tree and were found at the same location in the next morning.

The study was noninvasive and adhered to the Brazilian laws governing wild animal research.

### 2.4. Statistical Analysis

For statistical analysis, we used data of the 10 animals that had not disappeared during the study period. Data from adults and juveniles were analyzed together as juveniles of our study were ≥5 months old, a factor that makes their behavior fairly comparable to those found in adults [16, 49]. Each behavioral pattern was considered a discrete unit with a clear beginning and end ([45]; e.g., [13]). We estimated the frequency of a behavioral pattern by counting either the beginning or the end of the behavior [47]. The average duration of the observed behavioral patterns was short relative to the chosen session duration.

Spearman's correlation coefficient procedure was used to test if the frequency of a behavior correlated to the temperatures among time intervals. Thus, we correlated the mean maximum temperature for each time interval (*n* = 12) with the mean value of the observed behavioral frequency in relation to the number of sessions per individual in each time interval.

We used Friedman's test to verify if the frequency a given behavior (resting, foraging, locomotion, or gummivory) occurred across the time intervals varied significantly. As the number of sessions was slightly different among the individuals per time interval, we divided the total number of a certain behavior performed by a certain monkey by the number of sessions dedicated to that individual in every time interval. Dunn's post hoc test was applied to determine at what time interval the behavior increased or decreased significantly. Due to the small sample size, we excluded the following behavioral patterns from statistical analysis: grooming (7.6%), autogrooming (3.8%), and play (1.6%) behaviors.

For all analyses, the statistical significance was set at *P* ≤ 0.05. A unilateral test was employed when the results were predicted by a hypothesis; otherwise, bilateral tests were used [50]. Our data did not adhere to the parametric statistical model; thus we employed nonparametric tests [50]. All data were analyzed using the statistical program GraphPad InStat3 (GraphPad Software, Inc.) and Excel (Microsoft Corporation).

## 3. Results

### 3.1. Behavioral Data

Most of the observed behavioral patterns correlated significantly with temperatures among the time intervals. Thus, resting behavior increased significantly as temperature increased (Spearman's correlation coefficient: *n* = 12, *r*_*s*_ = 0.83, *T* = 4.62, and *P* = 0.0009). Locomotion behavior decreased as temperature increased (Spearman's correlation coefficient: *n* = 12, *r*_*s*_ = −0.78, *T* = −3.98, and *P* = 0.0026). Gummivory also decreased significantly as temperature increased. In addition, a negative significant correlation between gummivory and temperature was found (Spearman's correlation coefficient: *n* = 12, *r*_*s*_ = −0.60, *T* = −2.38, *P* = 0.0385). However, no significant correlation between foraging and temperature could be observed (Spearman's correlation coefficient: *n* = 12, *r*_*s*_ = −0.48, *T* = −1.74, and *P* > 0.05).

**Figure 2:**
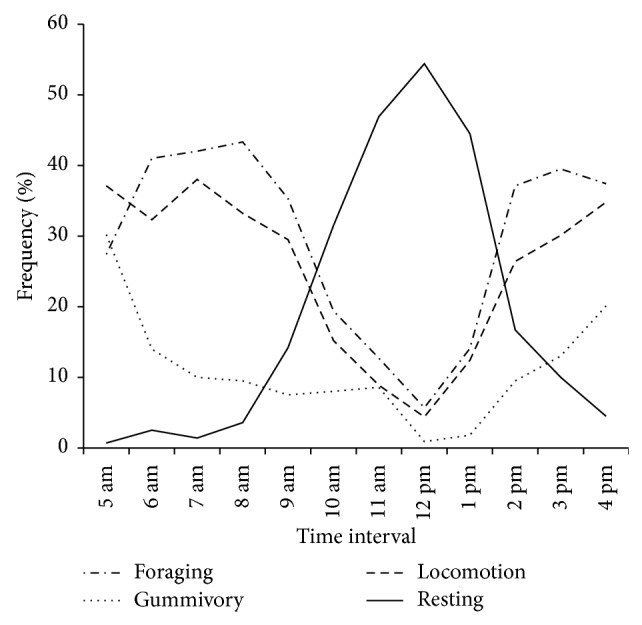
Behavioral activity among time intervals of wild common marmosets (*n* = 10) in the Caatinga.

The frequency of the observed behaviors also revealed significant differences among time intervals (Friedman's: foraging: *n* = 10, Fr = 74.93, df = 11, and *P* < 0.0001; gummivory: *n* = 10, Fr = 76.41, df = 11, and *P* < 0.0001; locomotion: *n* = 10, Fr = 81.87, df = 11, and *P* < 0.0001; resting: *n* = 10, Fr = 90.53, df = 11, and *P* < 0.0001). Foraging and locomotion were found to decrease significantly at 10 am (from 41% to 19.4% and 37.1% to 15.2%, resp.; Dunn's test: *P* ≤ 0.05), whereas gummivory exhibited a significant decrease after 8 am (from 30.2% to 9.5%; Dunn's test: *P* ≤ 0.05). Resting behavior increased significantly beginning from 10 am (from 0.7% to 31.5%; Dunn's test: *P* ≤ 0.05) and maintained a higher frequency (between 31.5% and 54.4% of all records; Dunn's test: *P* ≤ 0.05) until 2 pm, when it again decreased significantly (from 44.5% to 16.7%; Dunn's test: *P* ≤ 0.05). The decrease in resting behavior at 2 pm coincided with a significant increase in foraging (from 9% to 34%; Dunn's test: *P* ≤ 0.05) and locomotion (12.4% to 26.4%; Dunn's test: *P* ≤ 0.05). Gummivory significantly increased again at 3 pm (from 1.8% to 13%; Dunn's test: *P* ≤ 0.05). Foraging, gummivory, and locomotion were the most frequent behaviors until the animals went to the sleeping tree at approximately 5 pm (Figure 2).

### 3.2. Home Range and Intergroup Encounters

The average home range area of the two study groups was 2.73 ha (group A: 2.21 ha/group B: 3.26 ha) (Figure 1). We recorded intergroup encounters between group A and at least two other wild groups of common marmosets. The home range used by group B was located 540 m from the home range of group A. There were no other wild groups of common marmosets observed near the home range of group B over the study period.

### 3.3. Sleeping Cycle and Sleeping Sites

Study group A used seven sleeping sites and group B used six sleeping sites during the six-month study period. Neither group used the same sleeping site more than two or three nights in a row with a mean of 1.9 consecutive nights. All the sleeping sites were located in tree forks at the top of the highest trees (approx. mean height 5.72 ± SD 3.12 m) connected to the surrounding vegetation. Sleeping sites were situated in* P. juliflora *trees, with the exception of one sleeping site of group A located at the top of a coconut palm (*Cocos nucifera*, Arecaceae). In* P. juliflora*, the canopy was semicovered and the sleeping sites could be spotted among the leaves and branches of the trees. The sleeping site located on the coconut palm was completely covered, and the animals were hidden among the palm leaves. All group members slept together. The animals exited their sleeping sites, on average, 5 minutes (±SD 4.3 min) after sunrise and returned to their sleeping site approximately 15 minutes (±SD 9.8 min) before sunset (Table 3).

## 4. Discussion

Our findings show that common marmosets inhabiting the Caatinga adjusted their behavioral patterns to adapt to the high temperatures and apparent resource scarcity that characterize this environment. We base this assumption on the changes in behaviors such as resting, foraging, gum feeding, and locomotion that were observed with temperature fluctuations. Our results were comparable to those found in other primates living in similar environmental conditions (baboons: [27, 28]; vervet monkeys: [36]).

In our study, an association between temperature and resting was obtained throughout the day. These results complement each other and reinforce the importance of resting as a behavioral mechanism to avoid the risk of thermal stress (e.g., [28, 32]). While resting bouts in common marmosets at our study site occurred predominantly from 10 am to 2 pm, the same bouts were displayed in a much shorter interval (two hours) in the Atlantic Forest sites ([7, 8, 51]: 11 am–1 pm). Moreover, daily sleeping cycles also appeared to be longer in the Caatinga; that is, common marmosets left their sleeping site just five minutes after sunrise and returned to sleep only 15 minutes before sunset. These values are strikingly shorter when compared to those obtained in the Atlantic Forest conditions: 30 min after sunrise and the same length before sunset (e.g., [7, 8]). This behavior in the Caatinga might be a possible strategy to avoid the increase in body temperature, as the 10 am to 2 pm heat appears to reduce the frequency of locomotion and foraging activities.

In contrast to resting, which was displayed more frequently into the midday period (i.e., temperature becoming increasingly hot), foraging did not follow such a pattern. Instead, it maintained a level of occurrence up to ~10 am (~31°C) and then sharply decreased. As observed in vervet monkeys [36] in semiarid riparian woodlands, this result suggests that the Caatinga environment forces the small common marmosets to maintain the search for food for as long as they can thermally tolerate. To strengthen the idea of how important it is to obtain food in the Caatinga, locomotion was progressively reduced as temperature increased, whereas foraging did not decrease. That is, as the task of locomotion requires more physical effort from the common marmosets as temperature increased, they reduced this behavior however continued to search for food up to a certain point.

The need to compensate food scarcity also appeared to interfere in gummivory. Throughout the day, our study groups devoted an extensive period of time (up to 8 am) to this activity, especially during the morning hours. This also contrasts with data from Atlantic Forest sites (e.g., [8]: ~6 am to 7 am; ~4 pm to 5 pm). Such contrast suggests a high dependence on plant exudates by common marmosets living in the Caatinga, as gum is a readily available resource throughout the year. Amora et al. [19] observed that instead of relying mainly on gum exploitation, marmosets in the Caatinga used alternative food items such as leaves. This strategy was not observed during our study. Their diet consisted mainly of gum, fruits, arthropods, and small vertebrates, similar to the Atlantic Forest common marmosets [13]. It is still unclear how common marmosets adjust their diet toward food scarcity, and thus further studies need to be conducted in the Caatinga habitat.

Gummivory behavior by* C. jacchus *is also associated with the size of their home range [52]. In the home range of our study group, sizes were within the variation described for the Atlantic Forest ([51]: 2–5 ha; [53]: 0.72–1.62 ha; [7]: 0.5–3 ha; [8]: 4.98 ha; [54]: 2.5–6.5 ha; [55]: 4.11 ha). Apparently, the harsh condition such as in the Caatinga did not lead to a larger home range as we expected. The cost to explore a larger home range under high temperatures in such environment may exceed its benefits. However, intergroup encounters seemed to influence the home range size [54]. Thus, the smaller home range of group A might be explained, as this group had intergroup encounters with neighboring groups. By contrast, group B did not have any conflicts with other groups, which might have given them more freedom of movement to explore a larger area.

Finally, up to seven sleeping sites per group were used during our study period (6 months), and each site was not used more than three nights in a row. Previous reports on the number of sleeping sites in the Atlantic Forest areas vary considerably (e.g., [8]: *n* = 15 (11 months); [56]: *n* = 5 (10 months); [57]: *n* = 2 (20 months); [7]: *n* = 1–4 (4 months)). Sleeping sites in the Atlantic Forest are usually located in tall trees with a closed canopy [51, 57] and are near immediate sources of food such as gum [56]. In general, sleeping sites are selected to provide overall comfort and safety [58–62]. During our study period, sleeping sites were located in more open canopies. Nevertheless, animals chose to sleep nearby or even in the gum tree itself.

## 5. Conclusion

Overall, common marmosets inhabiting the Caatinga exhibited a number of behavioral adjustments toward temperature fluctuations. The extended period in which they display resting bouts particularly expresses the effect that high temperatures can have on this small primate. It is clear that the survival ability of these small primates in such a challenging environment can only be fully understood when the behavioral dimensions are taken into account. Our findings can help us better understand how common marmosets became one of the most successful primates, as well as how they colonize remarkably different environments such as the Caatinga and the Atlantic Forest.

## Figures and Tables

**Table 1:** Composition of the common marmosets groups in the study site.

Age class	Group A		Group B	
	♀	♂	♀	♂
Infant (1–4 months)	—	—	1^*^	1^*^
Juvenile (5–10 months)	—	3	1	1^*^
Adult (>11 months)	4 (2^*^)	1	2 (1^*^)	2

^*^Number of individuals that disappeared during the observation period. These individuals were not included in the statistical analyses.

**Table 2:** Description of the recorded behavioral patterns.

Behavior	Description
Resting	Individual is lying down on its belly or seated with its tail around the body or between the legs; the eyes may be open or closed [26]; it stays in this posture for more than 60 seconds [12].

Grooming	One individual parts the fur of another with its hands and removes particles such as dirt and parasites using its mouth and/or hands [7].

Autogrooming	Individual removes particles from its own skin and fur using its mouth and/or hands (adapted [7]).

Locomotion	Set of actions where the animal is moving from one place to another at a distance greater than 3 m at one time [12], which includes walking, running, climbing, and jumping [26].

Foraging	Group of actions in which the individual seeks and consumes food items (plant or animal). For our study, we did not consider the exploration for exudates in this category [8].

Gummivory	Set of acts where the individual gnaws the tree bark with its teeth and either licks or eats the exudate flow. It usually includes scent-marking the area with the circumgenital region at the end of the procedure [26].

Play	Interaction between two or more group members involving a series of playful actions [26] including hide-and-seek, wrestling, body-bite, and chase.

**Table 3:** Sleeping cycle and number of sleeping sites used monthly by common marmosets in the study period.

Month	Mean time				Number of sleeping sites	
	Sunrise^*^	Leave sleeping site	Sunset^*^	Return to sleeping site	Group A	Group B
Nov.	4:57	5:07	17:27	17:14	4	5
Dec.	5:03	5:14	17:36	17:11	2	4
Jan.	5:23	5:24	17:51	17:31	3	3
Feb.	5:29	5:31	17:48	17:24	4	3
Mar.	5:29	5:33	17:34	17:29	3	4
Apr.	5:27	5:31	17:21	17:19	2	3

Mean	5:18	5:23	17:36	17:21	3	3.7

^*^Source: http://euler.on.br/ephemeris/index.php.
